# Fully endoscopic access and resection of hemorrhaged cavernous malformation of the posterior midbrain

**DOI:** 10.3171/2019.7.FocusVid.1950

**Published:** 2019-07-01

**Authors:** Giulio Cecchini, Giovanni Vitale, Thomas J. Sorenson, Francesco Di Biase

**Affiliations:** 1 Department of Neurologic Surgery, San Carlo Hospital, Potenza, Italy;; 2 Department of Neurologic Surgery, Mayo Clinic, Rochester; and; 3 School of Medicine, University of Minnesota, Minneapolis, Minnesota

**Keywords:** endoscopy, brainstem, infratemporal approach, cavernous malformation, midbrain, video

## Abstract

Cavernous malformations in the midbrain can be accessed via several safe entry zones. The accepted rule of thumb is to enter at the point where the lesion is visible at the surface of the brainstem to pass through as little normal brain tissue as possible. However, in some cases, in order to avoid critical neural structures, this rule may not apply. A different safe entry zone can be chosen. Our video presents a case of a ruptured cavernous malformation in the midbrain reaching its anterior surface which was successfully resected via a posterolateral route using the supracerebellar infratentorial approach.

The video can be found here: https://youtu.be/j7VTqRO7qd4.

## Transcript

00:28 In this video, we present the case of a 40-year-old woman with a family history of cavernous malformations and recurrent hemorrhages. The patient was treated 2 months after her most recent hemorrhagic episode and was neurologically intact at time of treatment.

00:46 The MRI showed a brainstem lesion with the typical appearance of a cavernous malformation with hemosiderin ring. The lesion was located at the junction between posterior mesencephalon and upper pons, with about two-thirds in the supratentorial compartment and one-third in the infratentorial area. Preoperative angiography showed a draining vein running along the lateral surface of the posterior temporal lobe and draining then into the superior petrosal sinus. Because of the location of the lesion and this large draining vein, a fully endoscopic subtemporal approach was planned. While a conventional microscopic subtemporal approach would allow access to the midbrain by a similar route, in our experience, the endoscopic technique takes advantage of the possibility of having a deeper working angle from a similar craniotomy in order to access both the lateral and posterolateral part of the midbrain. Furthermore, we find that there is no need for retraction of the cerebrum because the superficial working tunnel is minimal and only a small corridor is needed for the surgical instruments, so the endoscopic vision can be just a few millimeters to the target. A lumbar drain was applied, and the patient was placed in a lateral position with the head gently tilted to the floor. Neuronavigation was used throughout the procedure.

02:24 A small linear incision was done over the ear and a 2.5-cm craniotomy over the temporal squama was performed, close to the floor of the middle cranial fossa.

02:39 After dural opening, infratemporal dissection was started with a four-handed fully endoscopic technique. The tentorial incisura was reached, and the arachnoid layers were gently opened in order to enter the crural and ambient cistern. The superior cerebellar artery and the trochlear nerve were identified.

03:22 The lesion became visible and a gentle bipolar coagulation was used in order to open its blood-filled spaces for decompression. Dissection started with suction in the left hand and a grasping microforceps in the right hand. With the forceps stabilizing the cavernous malformation, the suction gently detaches it from the parenchyma of the brainstem.

04:31 Holding the endoscope with a free hand allows us to have a perception of its depth and to have a dynamic view of the operating area. The use of dedicated endoscopic microinstruments is mandatory to have enough space for maneuvering within the narrow corridor. Further inspection with the endoscope showed a residual lesion inferiorly, towards the pons. This part was removed as well with a same technique. Hemostasis was achieved with fibrillin agents. The dura was sutured with watertight closure, and the bone flap was applied with titanium screws and plates.

06:18 MRI on postop day 3 showed complete removal of the lesion. The patient tolerated the procedure well with no new postoperative neurological deficit. She was discharged home on postoperative day 5.
